# The Perceptions of Physicians and Nurses Regarding the Establishment of Patient Safety in a Regional Teaching Hospital in Taiwan

**Published:** 2018-06

**Authors:** Chih-Hsuan HUANG, Hsin-Hung WU, Cindy Yunhsin CHOU, Huayong DAI, Yii-Ching LEE

**Affiliations:** 1. School of Business Administration, Hubei University of Economics, Wuhan, China; 2. Institute for Development of Cross-Strait Small and Medium Enterprise, Wuhan, China; 3. Dept. of Business Administration, National Changhua University of Education, Changhua, Taiwan; 4. Dept. of M-Commerce and Multimedia Applications, Asia University, Taichung, Taiwan; 5. College of Management, Yuan Ze University, Taoyuan, Taiwan; 6. Chung Kang Branch, Cheng Ching General Hospital, Taichung, Taiwan; 7. Dept. of Health Business Administration, Hung Kuang University, Taichung, Taiwan; 8. School of Health Policy and Management, Chung Shan Medical University, Taichung, Taiwan

**Keywords:** Patient safety, Organizational culture, Safety attitude questionnaire, Confirmatory factor analysis, Pearson correlation analysis

## Abstract

**Background:**

Physicians and nurses are core staff who are the first points of contact in care provision to patients. We aimed to examine physicians’ and nurses’ perceptions of patient safety in a case hospital by administering the Chinese Safety Attitude Questionnaire (CSAQ), developed from the Taiwan Joint Commission on Hospital Accreditation, in order to provide the patients with a safe environment and excellent medical service.

**Methods:**

An intra-organizational online survey was conducted. Confirmatory factor analysis (CFA) was first performed on the assessment of the measures for patient safety culture and seven dimensions with 33 items were identified. Pearson correlation analysis was subsequently used to examine the strength and direction of the relationships between seven dimensions of patient safety culture.

**Results:**

A total of 800 questionnaires were issued and 405 valid questionnaires were collected, the effective response rate being 50.6%. The findings highlighted that safety climate (SC) was positively and significantly related to teamwork climate (TC) and perception of management (PM), whereas stress recognition (SR) was negatively related to burnout (BUR).

**Conclusion:**

Patient safety culture in healthcare organizations has been considered a critical issue for improving the quality of healthcare. This study further focused on the contribution of a better patient safety culture for healthcare organizations in Taiwan.

## Introduction

Nowadays, the health care business has been increasingly competitive, and establishing a patient-oriented culture has become important for improving the safety and rights of patients in health care organizations in order to achieve sustainable development. It has proven that patient safety culture is a significant key influencing factor of improving physician-patient relationship and the service quality that patients receive (1). A positive safety culture allows healthcare employees to reduce medical errors or adverse events and learn from those failures (2, 3). The Safety Attitudes Questionnaire (SAQ) developed by Sexton et al. (4) has been widely adopted to assess patient safety culture for healthcare organizations (5, 6). Based on Sexton et al.’s (4) SAQ, the Chinese Safety Attitudes Questionnaire (CSAQ) developed by Taiwan Joint Commission on Hospital Accreditation (TJCHA) is a tool for hospitals to evaluate patient safety culture in Taiwan (7). Hospital managers can understand the perceptions of staff toward patient safety and further develop a scheme to achieve a superior physician-patient relationship in a competitive healthcare-based industry by using the questionnaire.

Patient safety culture was presented by the European Network for Patient Safety (EUNetPaS) in 2006 as “An integrated pattern of individual and organizational behavior, based upon shared beliefs and values that continuously seeks to minimize patient harm, which may result from the processes of care delivery” (8). Patient safety culture in healthcare organizations has received increasing attention (9, 10). Management’s commitment to patient safety can be realized by evaluating the attitudes of healthcare staff and patients on a regular basis (11, 12). Several studies have stated that healthcare staff with a positive attitude towards patient safety are more likely to reduce adverse events, including fewer medication errors (13–16), patient failures (14, 17, 18), urinary tract infections (13, 17, 19) and lower mortality (17, 18).

Hospital staff are on the front line of efforts to provide optimal care to patients. Lee et al. (20) particularly emphasized that the core staff are physicians and nurses. In fact, nurses are the biggest workforce in healthcare organizations and should have enough power to create a patient-oriented safety culture (21). Thus, it is critically important to understand opinions and attitudes of physicians and nurses toward patient safety, in order to provide the patients with a safe environment and excellent service experience.

In general, the SAQ contains 30 items, grounded into six dimensions: teamwork climate (TC), safety climate (SC), job satisfaction (JS), stress recognition (SR), perception of management (PM), and working conditions (WC). TC describes relationships and cooperation amongst staff members; SC refers to the perception of organizational commitment to patient safety; JS refers to positive opinions about the work experience; SR is related to the stress factors that might influence work performance; PM refers to management or administration approval; finally, WC is based on the perceived work-environment quality and logistical support regarding staffing and equipment (4, 21, 22).

In 2014, the TJCHA modified the questionnaire and integrated burnout (BUR) and work-life balance (WB), which contain nine and seven question items, respectively. BUR describes a loss of passion for work, emotional exhaustion and feelings of depersonalization (23) whereas WB refers to balancing both work and other aspects of life (24, 25). Consequently, the final form of the CSAQ consists of eight dimensions and 46 questions.

Reviewing patient safety culture on a regular basis enables hospital management to realize and monitor the progress of patient safety within the healthcare processes. Additionally, patient safety culture should be assessed periodically and contextually, because elements affecting patient safety culture might be different in different countries.

Consequently, the current research aimed to investigate the perceptions of hospital staff toward patient safety culture by using the CSAQ in order to establish a better patient safety culture for hospitals in Taiwan. Following this introduction, the second section of this paper illustrates details of the methods used in the empirical investigation; the third section presents the research results; implications are discussed in section four; and finally, the findings are concluded.

## Materials and Methods

### Study design and sample

An online-based survey design was conducted to investigate patent safety culture in a case hospital. The hospital contains more than 35 divisions, has 1500 total staff members, and provides clinical education and training to health professionals in Taichung City, Taiwan. An intra-organizational online survey with simple random sampling was conducted at the hospital; a total of 800 questionnaires were issued in 2014, of which 405 valid questionnaires were collected.

### Data collection

The surveys were sent to physicians and nurses between November 3 and November 24, 2014 in the case hospital. Approximately two weeks later, a reminder e-mail was sent to the subjects.

### Ethical aspects

This study was approved by the Institutional Review Board (IRB) of the general hospital (approval no. HP130026). Hospital staff completed an online-based survey. Consent was implied by the respondents’ participation in the study.

### Measurements

As shown in Table 1, the CSAQ was used to assess the perceptions of physicians and nurses about patient safety in the selected regional hospital. Seven patient safety dimensions with 39 items were measured except WB, since its scales were designed using frequency data, which are different from an interval scale. Therefore, physicians and nurses were asked to evaluate each question using a five-point Likert-type scale, where 1 and 5 represented strongly disagree and strongly agree, respectively. In addition, burnout was measured using nine reversed questions such that each respondent’s answer was adjusted.

**Table 1:** The Chinese safety attitude questionnaire

***TC***
1) Nurse input is well received in this clinical area 2) In this clinical area, it is difficult to speak up if I perceive a problem with patient care^(r)^ 3) Disagreements in this clinical area are resolved appropriately (i.e. not who is right, but what is best for the patient) 4) I have the support I need from other personnel to care for patients 5) It is easy for personnel here to ask questions when there is something that they do not understand 6) The physicians and nurses here work together as a well-coordinated team SC 7) I would feel safe being treated here as a patient 8) Medical errors are handled appropriately in this clinical area 9) I know the proper channels to direct questions regarding patient safety in this clinical area 10) I receive appropriate feedback about my performance 11) In this clinical area, it is difficult to discuss errors^(r)^ 12) I am encouraged by my colleagues to report any patient safety concerns I may have 13) The culture in this clinical area makes it easy to learn from the errors of others JS 14) I like my job (15) Working here is like being part of a large family 16) This is a good place to work 17) I am proud to work in this clinical area 18) Morale in this clinical area is high Stress Recognition 19) When my workload becomes excessive, my performance is impaired 20) I am less effective at work when fatigued 21) I am more likely to make errors in tense or hostile situations (e.g. emergency resuscitation, seizure) 22) Fatigue impairs my performance during emergency situations PM 23) Managers supports my daily efforts 24) Managers do not knowingly compromise patient safety 25) I get adequate, timely information about events that might affect my work 26) The levels of staffing in this clinical area are sufficient to handle the number of patients WC 27) Problem personnel are dealt with constructively 28) This hospital does a good job of training new personnel 29) All the necessary information for diagnostic and therapeutic decisions is routinely available to me 30) Trainees in my discipline are adequately supervised BUR 31) I feel like I’m at the end of my rope^(r)^ 32) I feel burned out from my work^(r)^ 33) I feel frustrated by my job^(r)^ 34) I feel I’m working too hard on my job^(r)^ 35) I feel emotionally drained from my work^(r)^ 36) I feel used up at the end of the workday^(r)^ 37) I feel fatigued when I get up in the morning and have to face another day on the job^(r)^ 38) Working with people all day is really a strain for me^(r)^ 39) Working with people directly puts too much stress on me^(r)^ WB 40) Missed meals 41) A hasty meal 42) All-day work without any rest 43) Individual or family plan change due to work factors 44) Poor sleep 45) Less than five-hour sleep at night 46) Late work

Note: TC: teamwork climate; SC: safety climate; JS: job satisfaction; SR: stress recognition; PM: perception of management; WC: working conditions; BUR: burnout; WB: work-life balance; r: reversed question

## Results

### Sample description

Analysis of the sample profile showed that most respondents were female (86.7%), their age ranging from 21 to 50 years (93.6%), and were educated at the bachelor’s level (92.1%). Over one-half of respondents had more than five years of relevant work experience for the hospital.

### Data analysis

The Kolmogorov–Smirnov test was first used for the estimation of normality of data. All variables followed the normal distribution at a significance level of .05. Additionally, in order to ensure that no offending estimate affected the loading of the items, the offending estimates were initially calculated before conducting confirmatory factor analysis (CFA). The common forms of offending estimates that appear in measurement were negative error variance, standardized loading of the manifest variables exceeding one, and high standard errors for the estimate coefficients. Seven dimensions with 39 items were estimated to confirm the structure of the data.

As shown in Table 2, there were no negative error variances, no standardized loadings exceeding one, and low standard errors. Thus, there were no offending estimates of the data.

**Table 2:** The results of offending estimates for items

***Variable***	***Means***	***SD***	***Error Variance***	***Standardized Loading***	***Standard Errors***
TC 1	3.42	.976	.329	.753	.038
TC 2^a^	3.83	.990	.813	.410	.058
TC 3	3.64	1.017	.316	.831	.034
TC 4	3.67	.979	.265	.796	.029
TC 5	3.96	.932	.215	.813	.025
TC 6	3.81	.967	.322	.842	.029
SC 1	3.58	1.035	.420	.779	.033
SC 2	3.67	.930	.203	.874	.018
SC 3	3.79	.860	.235	.826	.019
SC 4	3.44	.954	.356	.780	.028
SC 5^a^	3.44	.938	.719	.425	.051
SC 6	3.61	.882	.386	.709	.029
SC 7^a^	3.52	.913	.474	.656	.035
JS 1	3.49	.966	.364	.780	.028
JS 2	3.64	.961	.222	.871	.018
JS 3	3.58	.986	.158	.915	.015
JS 4	3.59	.931	.095	.944	.011
JS 5	3.41	.944	.281	.827	.022
SR 1	3.85	.989	.319	.820	.027
SR 2	3.87	1.040	.243	.881	.024
SR 3	3.61	1.027	.296	.848	.026
SR 4	3.75	1.046	.184	.912	.021
PM 1	3.48	.880	.222	.848	.019
PM 2	3.77	.974	.360	.787	.028
PM 3	3.56	.878	.141	.904	.015
PM 4^a^	2.85	1.109	.885	.527	.064
WC 1	3.36	.925	.276	.823	.023
WC 2	3.32	.946	.346	.783	.027
WC 3	3.55	.854	.154	.887	.015
WC 4	3.57	.849	.154	.886	.015
BUR 1	3.08	.923	.439	.700	.033
BUR 2	2.80	.959	.250	.853	.021
BUR 3	3.06	.903	.310	.787	.024
BUR 4^a^	2.68	.879	.427	.668	.032
BUR 5	2.79	.964	.218	.875	.020
BUR 6	2.46	1.025	.506	.719	.038
BUR 7	2.82	.997	.273	.851	.023
BUR 8	3.30	.954	.460	.702	.035
BUR 9^a^	3.39	.918	.521	.616	.038

Note: TC: teamwork climate; SC: safety climate; JS: job satisfaction; SR: stress recognition; PM: perception of management; WC: working conditions; BUR: burnout;

a: item deleted in subsequent confirmatory factor analysis; SD: standard deviation

CFA was then used to verify CASQ. A model re-specification was applied by confirming measurement items. Items with a weak factor loading (< .70) were removed from the original scale because of an elevated measurement error. In this stage, the six items (TC2, SC5, SC7, PM4, BUR4, and BUR9) shown in Table 2 were removed to improve the model fit for data. As a result, 33 items measuring seven dimensions were identified.

### Reliability and validity of instruments

All the Cronbach’s alpha coefficients are range between .88 (TC and PM) and .94 (JS). Convergent validity and discriminant validity were respectively assessed to confirm the dimensionality of patient safety culture and that the dimensions are distinct from each other. As presented in Table 3, the convergent validity results reveal that the composite reliability (CR) of all scales are above .90. Average variance extracted (AVE) estimates are range between .65 (SC) and .77 (JS).

**Table 3:** CFA results for seven dimensions of patient safety

***Dimensions***	***Mean***	***SD***	***Cronbach’s α***	***CR***	***AVE***	***Items***
1. TC	3.70	.7997	.88	.91	.69	5
2. SC	3.62	.7787	.89	.90	.65	5
3. JS	3.54	.8557	.94	.94	.77	5
4. SR	3.77	.9236	.92	.91	.71	4
5. PM	3.61	.8162	.88	.90	.75	3
6. WC	3.45	.7897	.91	.91	.72	4
7. BUR	2.77	.8000	.92	.92	.67	7

Note: TC: teamwork climate; SC: safety climate; JS: job satisfaction; SR: stress recognition; PM: perception of management; WC: working conditions; BUR: burnout; SD: standard deviation

Additionally, discriminant validity was demonstrated by comparing the AVE of each measure with the square of correlations between dimensions. As shown in Table 4, the AVE for all dimensions was greater than the square of the interfactor correlations between any two of each of the seven dimensions, thereby supporting the discriminant validity of the measures.

**Table 4:** Pearson’s correlations and squares of interfactor correlations for dimensions

	***1***	***2***	***3***	***4***	***5***	***6***	***7***
1.TC	.69						
2.SC	ϕ=.824^**^	.65					
	ϕ^2^=.678						
3.JS	ϕ=.740^**^	ϕ=.770^**^	.77				
	ϕ^2^=.495	ϕ^2^=.592					
4.SR	ϕ=.220^**^	ϕ=.193^**^	ϕ=.153^**^	.71			
	ϕ^2^=.048	ϕ^2^=.037	ϕ^2^=.023				
5.PM	ϕ=.738^**^	ϕ=.815^**^	ϕ=.768^**^	ϕ=.290	.75		
	ϕ^2^=.544	ϕ^2^=.664	ϕ^2^=.589	ϕ^2^=.084			
6.WC	ϕ=.714^**^	ϕ=.792^**^	ϕ=.726^**^	ϕ=.220^**^	ϕ=.818^**^	.72	
	ϕ^2^=.509	ϕ^2^=.627	ϕ^2^=.527	ϕ^2^=.048	ϕ^2^=.669		
7.BUR	ϕ=.240^**^	ϕ=.300^**^	ϕ=.389^**^	ϕ=−.223^**^	ϕ=.261^**^	ϕ=.304^**^	.67
	ϕ^2^=.057	ϕ^2^=.090	ϕ^2^=.151	ϕ^2^=.049	ϕ^2^=.068	ϕ^2^=.092	

Note: TC: teamwork climate; SC: safety climate; JS: job satisfaction; SR: stress recognition; PM: perception of management; WC: working conditions; BUR: burnout; ϕ: interfactor correlations; ϕ^2^: squares of interfactor correlations;

**: *P* < .01

The CFA results required the removal of six that had the estimated loadings lower than .70. Eventually, seven dimensions with 33 items were assessed to confirm the dimensionality of the scale. Furthermore, overall model fit was assessed using five absolute fit indices: chi-square (χ^2^), goodness of fit index (GFI), adjusted goodness of fit index (AGFI), root mean square residual (RMR), and root mean squared error of approximation (RMSEA); five incremental fit indices: normal fit index (NFI), Tucker Lewis index (TLI), relative fit index (RFI), comparative fit index (CFI), and incremental fit index (IFI); and two parsimonious fit indices: parsimony normed fit index (PNFI) and parsimony goodness of fit index (PGFI). The CFA results indicate that all fit indices’ values are within their threshold values, indicating a satisfactory goodness of fit for the measurement model to the data. For example, acceptable absolute fit indices (χ^2^/df = 2.235, GFI = .90, RMR = .07, and RMSEA = .05), incremental fit indices (TLI = .92, CFI = .95, and IFI = .92), and parsimonious fit indices (PNFI = .85 and PGFI = .76) are demonstrated.

### Results related to patient safety culture

To evaluate the overall essential dimensions for patient safety culture, the average values are provided in Table 3. TC has the highest average value followed by SC, whereas BUR has the lowest average value followed by WC.

Pearson’s correlation analysis was conducted to examine the strength and direction of the relationships among the seven dimensions of patient safety culture. As seen in Table 4, the results demonstrate that SC is positively and significantly related to TC and PM, as is PM to WC. On the other hand, SR is negatively related to BUR.

## Discussion

Establishing a positive patient safety culture has become a critical goal for healthcare organizations. The current study aimed to realize the perceptions of patient safety from physicians and nurses to create a better patient safety culture for healthcare organizations by examining the Chinese Safety Attitude Questionnaire.

First, the results of our study are consistent with those of Pinheiro and Uva (26), and Abdou and Saber (21), who suggested that an increase in the degree of TC improves SC, and that SC was highly correlated with PM. Good teamwork leads to positive attitudes toward patient care and happy team members (27), and less adverse medical errors (28, 29). In the same manner, it appeared likely that hospital staff would exhibit a greater cooperation if more resources and support for patient safety are provided by the hospital (14, 18).

Second, our results illustrate that PM is significantly related to WC (5), and has the second lowest average value among the seven dimensions. In fact, physicians and nurses typically work in a high-pressure environment that requires them to deal with difficult patient safety issues and complaints of their own experiences. Support and encouragement for physicians and nurses from hospital managers is important for ensuring patient safety, because it reflects the commitments and actions of hospital management (14). The current study suggests that hospital managers should develop systemic personnel training courses to assist physicians and nurses in recognizing patient safety issues.

Third, SR is negatively related to BUR. In other words, as the BUR of physicians and nurses increases, their SR decreases. The working environments of physicians and nurses normally encompass a wide range of potential stressors and challenges (23), and thus influence their work performance and emotional intelligence. With these concerns, this study suggests that appropriate management programs are needed to relieve such pressures. For example, employee assistance programs (EAP) can monitor the possible factors (such as stress management, interpersonal communication, and work adaptation) that might lead to a decrease in employee productivity (30).

## Conclusion

Patient safety culture has become a critical issue for healthcare organizations in improving management practices. It is extremely important to assess attitudes of physicians and nurses toward patient safety since they are on the front line of efforts to satisfy patients. Hospital managers should focus on enhancing the atmosphere of teamwork climate and safety climate in the hospital. A statement of support and commitment from the hospital manager, and humanized working conditions for physicians and nurses also play an important role in creating patient safety culture.

## Ethical considerations

Ethical issues (Including plagiarism, informed consent, misconduct, data fabrication and/or falsification, double publication and/or submission, redundancy, etc.) have been completely observed by the authors.
